# Opportunities for improving triple‐negative breast cancer outcomes: results of a population‐based study

**DOI:** 10.1002/cam4.998

**Published:** 2017-02-17

**Authors:** Elisabetta Rapiti, Kim Pinaud, Pierre O. Chappuis, Valeria Viassolo, Aurélie Ayme, Isabelle Neyroud‐Caspar, Massimo Usel, Christine Bouchardy

**Affiliations:** ^1^ Geneva Cancer RegistryUniversity of GenevaGenevaSwitzerland; ^2^ Oncogenetics and Cancer Prevention UnitDivision of OncologyGeneva University HospitalsGenevaSwitzerland; ^3^ Division of Genetic MedicineGeneva University HospitalsGenevaSwitzerland; ^4^ Molecular Clinical Pathology UnitDivision of Clinical PathologyGeneva University HospitalsGenevaSwitzerland

**Keywords:** Chemotherapy, genetic counseling, population‐based study, survival, triple‐negative breast cancer

## Abstract

Triple‐negative breast cancer (TNBC) is associated with a poor prognosis. Surgery, radiotherapy, chemotherapy, and referral for genetic counseling are the standard of care. We assessed TNBC prevalence, management, and outcome using data from the population‐based Geneva cancer registry. 2591 women had a first invasive stage I‐III breast cancer diagnosed between 2003 and 2011. We compared TNBC to other breast cancers (OBC) by *χ* ^2^‐test and logistic regression. Kaplan–Meier survival curves, up to 31‐12‐2014, were compared using log‐rank test. TNBC risk of mortality overall (OS) and for breast cancer (BCSS) was evaluated through Cox models. Linkage with the Oncogenetics and Cancer Prevention Unit (OCPU) database of the Geneva University Hospitals provided genetic counseling information. TNBC patients (*n* = 192, 7.4%) were younger, more often born in Africa or Central‐South America than OBC, had larger and more advanced tumors. 18% of TNBC patients did not receive chemotherapy. Thirty‐one (17%) TNBC women consulted the OCPU, 39% among those aged <40 years. Ten‐year survival was lower in TNBC than OBC (72% vs. 82% for BCSS;* P* < 0.001; 80% vs. 91% for OS;* P* < 0.001). The mortality risks remained significant after adjustment for other prognostic variables. The strongest determinants of mortality were age, place of birth, and lymph node status. A substantial proportion of TNBC patients in Geneva did not receive optimal care. Over 60% of eligible women did not receive genetic counseling and 18% did not receive chemotherapy. To improve TNBC prognosis, comprehensive care as recommended by standard guidelines should be offered to all patients.

## Introduction

Different subtypes of breast cancer can be distinguished according to the expression of biological markers in the primary tumor, some of them associated with a better prognosis than others. Triple‐negative breast cancer (TNBC) is one of the subtypes associated with a poor outcome 1, 2, 3. TNBCs do not express estrogen (ER) and progesterone receptors (PR), and do not overexpress and/or amplify human epidermal growth factor receptor‐2 (HER2), thus do not benefit from endocrine therapy or targeted immunotherapy (trastuzumab, pertuzumab).

In nonselected populations, TNBC represents 12–17% of all invasive breast cancer subtypes 4, 5 and are characterized by an aggressive pathological profile and poor clinical features. Several studies show a heterogeneity in TNBCs owing to different molecular alterations and/or supposed cells of origin (e.g., basal‐like and claudin‐low subtypes) 3, 6, 7.They usually have a high histologic grade and proliferation rate, occur often in younger women and in women of African and African American ancestry, have a shorter disease‐free survival time, and have a tendency to metastasize more often in visceral organs, including the central nervous system 4, 8, 9, 10. Up to 20% of women diagnosed with TNBC carry germline *BRCA1/BRCA2* mutations 11. This proportion varies according to the family history and the age at diagnosis, but remains at about 10% even in patients without relatives affected with breast or ovarian cancer 12. About 60–80% of breast cancers arising in carriers of constitutional *BRCA1* mutations show triple‐negative phenotype, whereas the rate of TNBC in BRCA2 carriers is closer to that observed in the general population 13, 14.

Management of patients with TNBC is challenging. Due to the lack of therapeutic targets, chemotherapy, both in the neoadjuvant and adjuvant settings, is the mainstay of systemic therapy 5. But the risk of recurrence and disease progression among these patients remains high 1. The possibility of disease causing germline *BRCA1/*BRCA2 mutations and the impact on surveillance and prevention measures, as well as family implications, requires considering these patients or genetic counseling.

Switzerland has a high incidence rate of breast cancer (83.1/100 000 in 2012) 15. However, no specific study on this breast cancer subtype has been conducted 16 and no information is available about management of TNBC in Switzerland. We investigated the prevalence, survival and potential factors affecting the outcome of TNBC in clinical practice using data from the population‐based Geneva Cancer Registry.

## Materials and Methods

The Geneva Cancer Registry records all incident cancers occurring in the population of the Geneva canton (450 000 inhabitants) since 1970. Hospitals, pathology laboratories, and private practitioners in the canton are requested to report all cancer cases. Trained tumor registrars systematically abstract data from medical and laboratory records. Physicians regularly receive enquiry forms to complete missing clinical and therapeutic data. The Registry regularly assesses survival taking as reference date the date of confirmation of diagnosis or the date of hospitalization (if it preceded the diagnosis and was related to the disease). Passive and active follow‐up is performed yearly using the files of the Cantonal Population Office (office in charge of the registration of the resident population). Cause of death is taken from clinical files.

As information about HER2 status has been recorded since 2003, for this study we considered all women resident in the Geneva area diagnosed with a first primary invasive breast cancer between 2003 and 2011 (*n = *3222). Cases discovered at autopsy (*n = *15), without histological confirmation (*n = *48), with unknown stage (*n = *76), women aged >85 years at diagnosis (*n = *156), breast lymphomas (*n = *3), metastatic cancers (*n = *138), and cases for whom one or more of the variables needed to define the TNBC status were missing (*n = *195) were excluded. The final cohort included 2591 women. According to the results of the ER, PR and HER2 tests we classified the women in two groups: women with <1% ER and PR values 17 and without overexpression of HER2 receptors were considered as having TNBC. The second group included all other types of breast cancer.

Variables considered in the analysis were age (<50, 50–69, or ≥70 years), birthplace (Switzerland, rest of Europe, Africa, North America, Central/South America, Asia/Oceania/Middle East, or unknown), socioeconomic status (SES) classified using the profession of the woman at the time of diagnosis or that of the husband for women without profession (high, medium, low, or unknown). The family history of breast or ovarian cancer was defined low for breast cancer patients without first‐ or second‐degree relatives with breast or ovarian cancer (i.e., sporadic cases); high, if patients reported one of the following family histories: (1) ≥1 first‐degree relative with breast or ovarian cancer ≤50 years; (2) ≥2 first‐degree relatives with breast/ovarian cancer at any age; (3) ≥3 cases of breast/ovarian cancer among first‐ or second‐degree relatives; and moderate for patients with other types of family history. The main sector providing care was classified as private versus public; the methods of detection of the breast tumor were screening, breast self‐examination, clinical examination, fortuitous, or unknown.

Tumors were classified according to the Tumor Node Metastasis (TNM) pathological system from the American Joint Committee on Cancer classification system 18 in stage I, II, or stage III. Other tumor characteristics considered were pathological tumor size in cm, lymph node status (positive, negative, or unknown), morphology (ductal, lobular, or other), multifocality (yes or no), synchronous in situ component (no, yes ductal, yes other, or unknown), and histological differentiation according to ICD‐O classification (well, moderately, poorly differentiated, or unknown).

Loco‐regional therapy was categorized as mastectomy, breast‐conserving surgery (BCS) followed by radiotherapy, other (including BCS without radiotherapy, or unknown surgery), or none. Use of radiotherapy was categorized as yes, no or unknown. Use of chemotherapy was categorized as neoadjuvant (± adjuvant), adjuvant, none, or unknown.

We opened the clinical files to determine why some patients with TNBC were not treated by chemotherapy (*n = *34).

A record linkage between the Geneva Cancer Registry database and that of the Oncogenetics and Cancer Prevention Unit (OCPU) of the Geneva University Hospitals provided information about use of genetic counseling and testing by these women. The OCPU has been set up in 1994 as a consultation center providing genetic counseling for familial aggregation of malignant tumors or hereditary cancer predisposition syndromes in the Geneva area. In case of personal or familial medical history suggestive of a hereditary cancer susceptibility syndrome, the possibility to undergo genetic testing is extensively discussed as part of the genetic counseling. Since this study did not involve primary care physicians/specialists managing breast cancer patients, information concerning the exact proportion of affected women referred to OCPU is not known, thus the rate of patients who choose not to undergo oncogenetic consultation could not be determined.

### Statistical methods

We compared TNBC patients with all the OBC patients for demographic, tumor, and treatment characteristics using univariate and multivariate logistic regression analysis, considering women with TNBC as cases and the OBC women as controls.

All women of the cohort were followed for vital status up to December 31, 2014. The overall survival (OS) and breast cancer‐specific survival (BCSS) were calculated by Kaplan–Meier method and the survival curves of TNBC and the OBC patients were compared through the log‐rank test. We evaluated the crude and adjusted effect of triple‐negative status on 10‐year OS and BCSS using Cox proportional hazards regression models.

Within the TNBC group, we evaluated the differences between women who consulted the OCPU and those who did not. Logistic regression models were performed to assess determinants of consultation.

Univariate and multivariate Cox models were used to assess determinants of overall mortality among women with TNBC. Variables entered in the multiadjusted model were those statistical significant in the univariate models.

A *P‐value* <0.05 was considered as statistically significant. All the analyses were performed using the IBM SPSS statistics software version 22.

## Results

Among the 2591 women included in the study, 192 (7.4%) had a TNBC. In Table 1, we present the socio‐demographic, clinical, and pathological characteristics of patients with TNBC and OBC subtypes and the results of the logistic regression models.

**Table 1:** Patient, clinical, and pathological characteristics according to breast cancer triple‐negative status. Odds ratios (OR) from logistic models

	Triple negative			Crude OR (95% CI)	Adjusted OR^b^ (95% CI)
	No (%) (*n = *2399)	Yes (%) (*n = *192)	*P‐value χ* ^2^ a		
Age category (years)			0.006		
<50	585 (24.4)	65 (33.9)		1	
50–69	1248 (52.0)	95 (49.5)		0.69 (0.49–0.95)*	
≥70	566 (23.6)	32 (16.7)		0.51 (0.33–0.79)**	
Birthplace			0.008		
Switzerland	1192 (49.9)	78 (41.1)		1	
Europe	917 (38.4)	73 (38.4)		1.22 (0.87–1.69)	
Africa	103 (4.3)	17 (8.9)		2.52 (1.44–4.42)**	
North America	35 (1.5)	5 (2.6)		2.18 (0.83–5.73)	
Central & S. America	71 (3.0)	10 (5.3)		2.15 (1.07–4.34)*	
Asia, Oceania, Mid. East	70 (2.9)	7 (3.7)		1.53 (0.68–3.44)	
Unknown	11	2		2.78 (0.61–12.8)	
Socioeconomic status			0.812		
High	535 (23.1)	45 (23.8)		1	
Medium	1342 (57.8)	105 (55.6)		0.93 (0.65–1.34)	
Low	443 (19.1)	39 (20.6)		1.05 (0.67–1.64)	
Unknown	79	3		0.45 (0.14–1.49)	
Family history			0.710		
Without	1465 (65.6)	126 (68.5)		1	
Moderate	594 (26.6)	44 (23.9)		0.86 (0.60–1.23)	
High	174 (7.8)	14 (7.6)		0.94 (0.53–1.66)	
Unknown	166	8		0.56 (0.27–1.17)	
Method of detection			<0.001		
Screening	1088 (46.3)	45 (23.6)		1	1
Self‐examination	785 (33.4)	104 (54.5)		3.20 (2.23–4.60)***	1.76 (1.17–2.64)**
Clinical exam	190 (8.1)	11 (5.8)		1.40 (0.71–2.76)	1.09 (0.53–2.25)
Fortuitous	288 (12.3)	31 (16.2)		2.60 (1.62–4.19)***	1.73 (1.00–2.98)*
Unknown	48	1		0.50 (0.07–3.73)	0.35 (0.04–2.74)
Sector of care			0.764		
Private	1208 (50.4)	94 (49.0)		1	
Public	1191 (49.6)	98 (51.0)		1.06 (0.79–1.42)	
Morphology			<0.001		
Ductal	1907 (79.5)	168 (87.5)		1	1
Lobular	396 (16.5)	7 (3.6)		0.20 (0.09–0.43)***	0.35 (0.15–0.82)*
Other	96 (4.0)	17 (8.9)		2.01 (1.17–3.45)*	1.72 (0.91–3.27)
Multifocality			0.007		
No	1749 (72.9)	157 (81.8)		1	1
Yes	650 (27.1)	35 (18.2)		0.60 (0.41–0.88)**	0.68 (0.45–1.04)
In situ component			<0.001		
No	666 (29.9)	73 (44.2)		1	1
Yes, ductal	1264 (56.8)	86 (52.1)		0.62 (0.45–0.86)**	0.58 (0.40–0.84)**
Yes, other	294 (13.2)	6 (3.6)		0.19 (0.08–0.43)***	0.36 (0.14–0.91)*
Unknown	175	27		1.41 (1.43–3.59)	0.46 (0.22–0.94)*
Stage			<0.001		
I	1133 (47.2)	58 (30.2)		1	
II	962 (40.1)	95 (49.5)		1.93 (1.38–2.70)***	
III	304 (12.7)	39 (20.3)		2.51 (1.64–3.83)***	
Axillary lymph node status			0.313		
Positive	881 (36.9)	78 (40.6)		1	1
Negative	1507 (63.1)	114 (59.4)		0.85 (0.63–1.15)	1.53 (1.06–2.19)*
Unknown	11	‐		–	–
Tumor size (cm)			<0.001		
<2	1401 (65.1)	72 (48.6)		1	1
<5	651 (30.2)	66 (44.6)		1.97 (1.40–2.79)***	1.33 (0.90–1.98)
≥5	101 (4.7)	10 (6.8)		1.93 (0.97–3.85)	2.18 (0.97–4.88)
Unknown	246	44		3.48 (2.34–5.19)***	2.69 (1.42–5.08)**
Differentiation			<0.001		
Well	647 (27.2)	9 (4.8)		1	1
Moderately	1349 (56.6)	47 (24.9)		2.51 (1.22–5.14)*	2.69 (1.30–5.57)**
Poorly	387 (16.2)	133 (70.4)		24.7 (12.4–49.1)***	21.3 (10.5–43.2)***
Unknown	16	3		13.5 (3.33–54.5)***	6.51 (1.48–28.6)*

a chi‐square for heterogeneity are calculated excluding unknown.

b The final model has been derived from a backward stepwise procedure starting with all the variables in the univariate model except stage because of its redundancy with pathological tumor size and axillary lymph node status.

*P*‐value *<0.05; **<0.01; ***<0.001.

### Characteristics of TNBC patients

Women with TNBC were younger than those with OBC and less likely to have been born in Europe (79.5% *vs*. 88.3%). TNBC patients were more often coming from Africa and Central and South America.

The TNBCs were more likely discovered through self‐examination or fortuitously, during a medical assessment or hospitalization for other health problems than OBCs (54.5% *vs*. 33.4% and 16.2% *vs*. 12.3%*,* respectively), and were less likely detected by screening (23.6% *vs*. 46.3%; *P* < 0.001).

TNBC tumors were larger at diagnosis than OBC tumors (51.4% *vs*. 34.9%, respectively, for tumor size >2.0 cm, *P < *0.001), more often ductal and without an in situ component than OBC tumors. More than 70% of TNBC tumors had a poorly differentiated histologic grade *v*ersus 16.2% among OBC (*P = *0.001), and were mostly associated with absence of multifocality (*P = *0.007). The proportions of stage II and stage III were 49.5% and 20.3% in the TNBC group versus 40.1% and 12.7% in the OBC group, respectively (*P* < 0.001). The two groups were similar in terms of axillary lymph node status (*P* = 0.313).

Variables included in the multivariate logistic regression model were selected through a backward stepwise procedure starting with a model with all the variables included. As we included pathological tumor size and lymph node status, stage was not included in the full model. In the final model, TNBC remained strongly associated with higher risk of being diagnosed through self‐examination or fortuitously, a ductal tumor morphology, without an in situ component, negative lymph nodes status, larger and moderately or poorly differentiated tumors.

### Treatment of TNBC

Table 2 shows treatments received by women with TNBC versus OBC subtypes. There were no significant differences between the two groups in terms of surgery (*P* = 0.443) and radiotherapy use (*P* = 0.066). Patients with TNBC were more frequently treated with both neoadjuvant and adjuvant chemotherapy than OBC patients(*P* < 0.001). The most frequent reasons for not receiving chemotherapy among TNBC cases were old age (*n = *6), refusal (*n = *8), low‐risk tumor characteristics (small size, absence of lymph node invasion, well differentiated tumors) (*n = *4), and presence of comorbidities (*n = *3). For 13 patients, the reasons they were not treated with chemotherapy were either unknown or discussed but not documented. Few patients with TNBC were treated by hormonotherapy (*n = *5). In one case, an oophorectomy was performed because of young age. For the other four women, a positive hormonal receptor status was found in a carcinoma affecting the contralateral breast, or in a previous biopsy, or after the chemotherapy.

**Table 2:** Treatment of breast cancer patients according to triple‐negative tumor status

	Triple‐negative breast cancer		*P‐*value^a^
	No (*n = *2399)	Yes (*n = *192)	
	*n* (%)	*n* (%)	
Chemotherapy			
Yes	850 (35.5)	157 (82.2)	<0.001^b^
Neoadjuvant	184 (7.7)	45 (23.6)	<0.001^c^
Adjuvant only	666 (27.8)	112 (58.6)	
No	1544 (64.5)	34 (17.8)	
Unknown	5	1	
Surgery			
Yes	2339 (97.5)	189 (98.4)	0.443^b^
BCS with radiotherapy	1627 (67.8)	129 (67.2)	0.173^d^
Mastectomy	559 (23.3)	53 (27.6)	
Other^e^	153 (6.4)	7 (3.6)	
No	60 (2.5)	3 (1.6)	
Radiotherapy			
Yes	1872 (80.2)	159 (85.9)	0.066
No	463 (19.8)	26 (14.1)	
Unknown	64	7	
Hormonotherapy			
Yes	1702 (80.5)	5 (2.6)	<0.001
No	411 (19.5)	185 (97.4)	
Unknown	286	2	

BCS, breast conservative surgery.

a *P‐value* calculated after exclusion of unknown.

b *P‐value* for chemotherapy or surgery categorized as yes/no.

c *P‐value* for chemotherapy yes: neoadjuvant *vs*. adjuvant only.

d *P‐value* for surgery yes: BCS with radiotherapy *vs*. mastectomy *vs*. other.

e The category other includes BCS without radiotherapy and surgery unknown.

### Genetic counseling in the Oncogenetics and Cancer Prevention Unit

Among the 192 TNBC women, 33 (17%) consulted the OCPU. Compared with women who did not consult those who consulted were younger than 40 years of age, more likely born in Europe or North America, had a stronger family history of breast or ovarian cancer, had earlier stage disease at diagnosis and more often had a poorly differentiated tumor. Although not significant, we also observed a trend toward an increase in consultation with the most recent period of diagnosis. In a multiadjusted logistic regression model chosen through back step procedure, with all variables except axillary lymph node status because of redundancy with stage, we found that variables independently associated with OCPU consultation among TNBC women included age younger than 40 years (Odds Ratio (OR) = 14.0; 95% CI:3.49–55.9), being born outside Europe or North America (OR for being born in other countries =0.09 (95% CI: 0.01–0.87), more recent period of diagnosis (OR for 2009–2011 = 4.80; 95% CI: 1.25–18.4), a moderate and high‐risk family history(OR=5.04; 95% CI:1.62–15.6 for moderate risk; OR=69.2; 95% CI:10.0–477 for high risk), being treated in the public sector (OR=4.05; 95% CI:1.12–14.5), and an early stage (OR for stage III=0.10; 95% CI:0.02–0.60).

Among the 33 women who consulted, 24 (73%) underwent germ‐line testing of *BRCA1/BRCA2* genes and 9 (37.5%) tested positive for a pathogenic variant.

### Impact of TNBC subtype on survival

**Figure 1:**
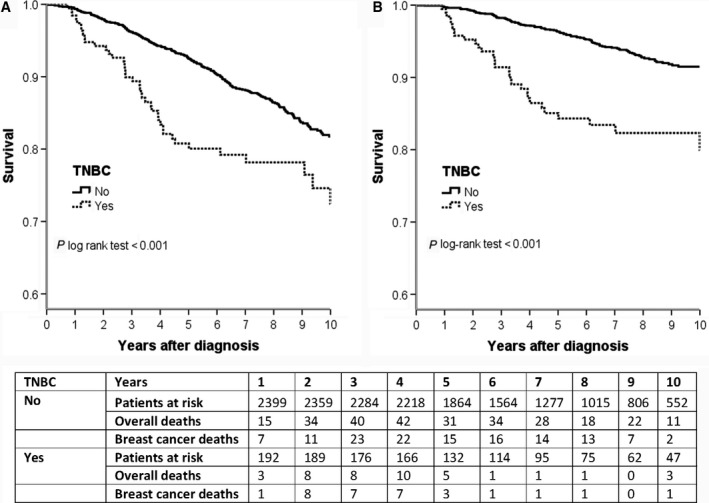
Overall survival (panel A) and breast cancer‐specific survival (panel B) of women with triple‐negative breast cancer (TNBC) and other breast cancer subtypes.

All women were followed on average for 6.5 years (median time for TNBC: 4.5 years; median time for OBC: 4.3 years). At the end of the follow‐up period, 41 women from the TNBC group were deceased (30 of breast cancer) and 283 from the OBC group (131 of breast cancer). The cause of death was unknown for 22 women. Compared with OBC patients, women with TNBC had a statistically significantly lower OS and BCSS (Fig. 1). The 10‐year survival estimates were 0.80 for TNBC patients (95% CI: 0.72–0.88) versus 0.91 for OBC patients (95% CI: 0.89–0.93) for all causes of death (*P* of log‐rank test <0.001) and 0.72 for TNBC patients (95% CI: 0.64–0.80) versus 0.82 for OBC patients (95% CI: 0.80–0.84) for death from breast cancer (*P* of log‐rank test <0.001). After approximately 5 years of follow‐up, the number of deaths due to breast cancer among the TNBC group started slowly to decline.

The mortality risk for TNBC *vs*. OBC patients was derived from the univariate and multivariate Cox regression models separately for overall and breast cancer mortality. In the univariate model, breast cancer mortality of patients with TNBC was three times higher and overall mortality almost two times higher than that of patients with OBC (Hazard ratio (HR) = 3.08, 95% CI: 2.07–4.57 and HR = 1.92, 95% CI: 1.38–2.68, respectively). After adjusting for other confounding and prognostic variables, such as age, pathological tumor size, lymph node status, multifocality, in situ component, differentiation, method of discovery, surgery and chemotherapy, women with TNBC had still an almost doubled risk of dying from breast cancer (HR = 1.86, 95% CI: 1.17–2.95), and a nonsignificant 50% higher risk of death from all causes (HR = 1.46, 95% CI: 0.99–2.15).

Table 3 presents the risk of dying from all causes among TNBC patients. The strongest independent determinants of overall mortality for TNBC patients from a multiadjusted Cox model were age ≥70 years (HR = 2.00, 95% CI: 1.00–4.88), being born in a country outside Europe or North America (HR = 2.40, 95% CI: 1.17–4.95), not being diagnosed by screening, and a positive lymph node status (HR for negative lymph node status = 0.47, 95% CI: 0.24–0.95).

**Table 3:** Risk of dying from all causes among women with TNBC according to clinical characteristics of the tumor and treatment

	Univariate	Multiadjusted model^a^
	HR (95% CI)	HR (95% CI)
Age category (years)		
<50	1	1
50–69	0.89 (0.43–1.82)	0.90 (0.41–1.99)
≥70	1.74 (0.78–3.88)	2.00 (1.00–4.88)
Birthplace		
Europe + North America	1	1
Other	2.22 (1.13–4.35)*	2.40 (1.17–4.95)*
Familial history		
Without	1	
Moderate	0.95 (0.46–1.94)	
High	0.53 (0.13–2.21)	
Unknown	–	
Method of detection		
Screening	1	1
Self‐examination	3.24 (1.13–9.31)*	2.49 (0.83–7.48)
Clinical exam	3.43 (0.77–15.3)	3.05 (0.65–14.4)
Fortuitous	4.06 (1.21–13.6)*	2.45 (0.68–8.84)
Unknown	–	–
Sector of care		
Private	1	
Public	1.18 (0.63–2.19)	
Genetic counseling consultation		
No	1	
Yes	0.49 (0.18–1.32)	
Histology		
Ductal	1	
Lobular	2.17 (0.67–7.05)	
Other	0.50 (0.12–2.10)	
Multifocality		
No	1	
Yes	1.64 (0.81–3.36)	
In situ component		
No	1	
Yes, ductal	1.88 (0.88–4.03)	
Yes, other	2.46 (0.54–11.3)	
Unknown	2.90 (1.18–7.14)	
Lymph nodes status		
Positive	1	1
Negative	0.39 (0.21–0.73)**	0.47 (0.24–0.95)*
Tumor size (cm)		
<2	1	1
<5	1.90 (0.81–4.45)	1.55 (0.74–3.76)
≥5	3.85 (1.18–12.5)*	2.51 (0.69–9.20)
Unknown	3.08 (1.35–7.03)**	1.76 (0.72–4.34)
Stage		
I	1	
II	1.97 (0.78–5.00)	
III	5.90 (2.34–14.9)***	
Differentiation		
Well/Moderately	1	1
Poorly	1.08 (0.53–2.17)	0.96 (0.45–2.06)
Unknown	4.93 (1.08–22.4)*	5.00 (0.92–27.0)
Chemotherapy		
Yes	1	
No	1.29 (0.62–2.70)	
Unknown	–	

HR, hazard ratio; CI, confidence interval.

*P*‐value *<0.05; **<0.01; ***<0.001.

a Model adjusted on: age, place of birth, method of detection, axillary lymph node status, pathological tumor size, and differentiation.

## Discussion

This is the first study comprehensively investigating TNBC characteristics and outcome in a population‐based setting in Switzerland. This type of breast cancer accounted for just over 7% of all breast cancers diagnosed in Geneva area between 2003 and 2011. The majority of these patients were treated according to international guidelines. However, a substantial proportion of these patients did not receive chemotherapy as recommended, and only 40% of the patients diagnosed before age 40 years had an oncogenetic evaluation. This study confirms that women with TNBC experienced a worse survival than women with OBC subtypes, even after adjusting for tumor characteristics and treatment. The excess of mortality due to breast cancer among TNBC women slowly started declining 5 years after diagnosis. Sociodemographic and tumor characteristics appeared to be the main determinants of TNBC survival.

TNBC has been associated with African ancestry and Hispanic race and its prevalence in the USA accounts for approximately 15% of all breast cancers 10, 19, 20. The proportion of TNBC in our female breast cancer population, largely represented by Caucasian patients, was lower but comparable with that of other European countries 16. In a recent population‐based study covering all women diagnosed with breast cancer in England in 2012–2013, Moller et al. found a proportion of TNBC of 5% and suggested that the lack of information data on receptors was likely to explain this low estimate 21. TNBC patients in our study were mostly detected by self‐examination or fortuitously. This is partially explained by the younger age at onset of TNBC women, 33% were <50 years old, therefore not included in the breast cancer screening program. Furthermore, several studies reported that TNBCs are more frequent among interval cancers, that is, cancers detected between screening intervals, than screen‐detected cancers 9, 22, 23. Gene expression profiling studies have shown that the majority of TNBCs falls into the basal‐like carcinoma molecular subtype 4, 24, 25 which is more frequently diagnosed as interval cancers 26.

TNBC presents more aggressive characteristics than OBC. In our population, only 30% of TNBC patients were diagnosed at stage I, and over 70% presented with poorly differentiated tumors, proportions very close to those observed by other authors 9. Interestingly, TNBCs were, in contrast, less likely to be multifocal and lymph node positive than OBCs, the latter finding suggesting a preferential hematogenous dissemination for the metastatic process 27.

Even though patients with TNBC were more frequently treated by chemotherapy than OBC patients, 18% of them did not receive chemotherapy in our cohort. Chemotherapy is so far considered as the only curative systemic treatment for TNBC 28. Among tumors displaying triple‐negative phenotype, only adenoid cystic, apocrine, or medullary carcinomas not necessarily require chemotherapy 29, as these subtypes have an exceptionally good prognosis; however, they are very rare. In our population, there were two adenoid cystic and four medullary carcinomas and three of them did not receive chemotherapy. Patient‐ and physician‐related factors are known to prevent patients from receiving standard treatments. By reviewing the clinical files, we found as reasons for not receiving chemotherapy supposed low‐risk cancer features (low grade and/or small size), refusal, presence of comorbidities and old age. However, it has been demonstrated that even in this subgroup of patients with favorable features adherence to guidelines in terms of chemotherapy and radiotherapy improves survival 30. The small size of our TNBC population and of the subgroup without chemotherapy did not provide sufficient statistical power to adequately assess the effect of chemotherapy on survival.

Another issue regarding compliance to standard protocols is the fact that only 17% of the whole TNBC population and 39% of those aged <40 years had a genetic counseling. In addition to young age at diagnosis, the other clinical information for referring patients to genetic counseling in our study was a positive family history. Recommendations to propose genetic counseling and *BRCA1/BRCA2* testing to women with TNBC regardless of family history is relatively recent, and was only recommended in 2003. The observed increase in the trend of using genetic counseling at OCPU by patients with TNBC in the most recent periods, and particularly in 2009–2011, likely reflects the inclusion of this recommendation in clinical practice. Concerning early onset breast cancer, this is a criterion stated as early as 1996^31^. In the absence of family history of breast or ovarian cancer, current guidelines are to propose genetic counseling and testing to all patients diagnosed with TNBC under age 60 years 32, 33; or diagnosed with any subtype of breast cancer under age 40 34, 35.

Most breast cancers arising in the setting of *BRCA1* germ‐line mutations are triple‐negative 13, but TNBCs are also observed among *BRCA2* mutation carriers 36, 37. Referral to an oncogenetic consultation and subsequent genetic testing influence treatment and follow‐up choices for breast cancer patients and their blood relatives. In fact, in individuals carrying *BRCA1*/*BRCA2* germ‐line mutations predisposing to breast and ovarian cancer, screening and prevention protocols have demonstrated their efficacy in terms of gain of life expectancy 38. In our population, more than two‐thirds of women who met the criteria for genetic testing did not consult the oncogenetic unit and therefore were not tested, as the OCPU is the only centre providing genetic counseling in the Geneva area. In a study conducted in the USA among young breast cancer survivors, Ruddy et al. found that only 24% of patients ≤40 years old at diagnosis underwent genetic testing 39. Similarly, from the same dataset, Brown et al. found that only 45% of 1221 women with early onset breast cancer ever discussed genetic testing and/or had been referred to a genetic counselor 31. A recent study aiming to determine referral patterns for genetic counseling in women who met NCCN guidelines between 2004 and 2010 found that only 34% of these women were actually referred, a proportion very close to that found in our study 40. As in our study, age and family history were the strongest predictive factors for referral 40.

TNBC patients in our study showed an almost doubled risk of dying from breast cancer compared with OBC patients, an estimate similar to that found in other studies 19, 21, 41. After an extremely high mortality rate during the first 5 years following diagnosis, the mortality curves of TNBC patients, both specific and overall, seem to level. Our finding is not isolated 1, 9, 19. A complete response to neoadjuvant treatment has been proposed as the main reason for this effect 1, whereas some authors have hypothesized that different neoadjuvant regimens may have a different impact on TNBC survival 42. Altogether, these results suggest the existence of some subtypes of TNBC that have a better prognosis and that may respond better to adjuvant chemotherapy. Unfortunately, in our database, we do not have detailed information on type of chemotherapy used and the number of patients who received neoadjuvant chemotherapy is too small to allow a stratified analysis.

One of the strongest determinants of OS in our population, besides the clinical characteristics of the tumor, was the place of birth. Women born outside Europe and North America represent around 10% of all breast cancer patients diagnosed in Geneva, but 18% of TNBC. These women showed a more than twofold increased risk of dying for all causes in the multiadjusted Cox model. The existence of racial disparities in breast cancer survival has largely been demonstrated and has been associated to socioeconomic differences, a different access to health care, as well as biological characteristics of breast cancer 43, 44. We observed that women born in Africa, Asia and South America were younger, had a more advanced stage at diagnosis and were less often seen in the OCPU. These women represent a group of breast cancer patients at high risk for a poor outcome, any barrier to an appropriate management of these women should be eliminated to improve their prognosis.

The limitations of this study are mainly related to its retrospective design. Our dataset does not include full information about ethnicity and type of chemotherapy. In addition, although our results are statistically significant, our TNBC population and follow‐up period are relatively small.

The current lack of targets for specific treatments and the strong association with *BRCA1/BRCA2* mutations make the management and treatment of TNBC patients rather challenging. Our study shows that although most of TNBC patients in the Geneva area were managed according to standard guidelines, a substantial proportion of them did not receive optimal care. There is opportunity for improving TNBC patients’ management. A prompt and complete evaluation of these TNBC patients, which includes, among others, the referral to an oncogenetic consultation and the delivery of comprehensive care according to standard guidelines, should be offered to all women to obtain the best possible results and improve their survival chances.

## Conflict of Interest

The authors declare no conflicts of interest.
